# Molecular Characteristics and Role of Buffalo *SREBF2* in Triglyceride and Cholesterol Biosynthesis in Mammary Epithelial Cells

**DOI:** 10.3390/genes16020237

**Published:** 2025-02-19

**Authors:** Wenbin Dao, Hongyan Chen, Yina Ouyang, Lige Huang, Xinyang Fan, Yongwang Miao

**Affiliations:** 1 Institute of Animal Genetics and Breeding, College of Animal Science and Technology, Yunnan Agricultural University, Kunming 650201, China; dwbin666@126.com (W.D.); 18838969693@163.com (L.H.); xinyangfan1@ynau.edu.cn (X.F.); 2 Faculty of Animal Husbandry and Veterinary Medicine, Yunnan Vocational College of Agriculture, Kunming 650212, China; chykmzjy@126.com; 3 Yunnan Institute of Animal Science and Veterinary, Kunming 650224, China; yinaouyang@126.com

**Keywords:** *SREBF2*, buffalo mammary epithelial cells, gene function, triglyceride, cholesterol synthesis

## Abstract

**Background/Objectives:** Sterol regulatory element-binding transcription factor 2 (SREBF2) is a key transcription factor involved in regulating cholesterol homeostasis. However, its role in buffalo mammary gland lipid metabolism remains unclear. **Methods**: To address this, we isolated and characterized the *SREBF2* gene from buffalo mammary glands and performed an in-depth analysis of its molecular characteristics, tissue-specific expression, and functional roles in buffalo mammary epithelial cells (BuMECs). Additionally, we investigated the single nucleotide polymorphisms (SNPs) of *SREBF2* in both river and swamp buffalo. **Results**: The coding sequence (CDS) of buffalo *SREBF2* is 3327 bp long and encodes a protein of 1108 amino acid residues. Bioinformatics analysis revealed that the molecular characteristics of buffalo *SREBF2* were highly similar across Bovidae species, with collinearity being observed among them. An expression profile analysis revealed that *SREBF2* is expressed in all 11 tested tissues of buffalo, with its expression level in the mammary gland being higher during lactation than in the dry period. The knockdown of *SREBF2* in BuMECs during lactation led to a significant reduction in the expression of genes involved in triglyceride (TAG) and cholesterol synthesis, including *PI3K*, *AKT*, *mTOR*, *SREBF1*, *PPARG*, *INSIG1*, *ACACA*, *SCD*, *DGAT1*, *LPL*, *CD36*, *HMGCR*, and *SQLE*. This knockdown led to a 23.53% and 94.56% reduction in TAG and cholesterol levels in BuMECs, respectively. In addition, a total of 22 SNPs were identified in both buffalo types, of which four non-synonymous substitutions (c.301G>C, c.304A>T, c.1240G>A, and c.2944G>A) were found exclusively in the *SREBF2* CDS of swamp buffalo, and the assessment revealed that these substitutions had no impact on *SREBF2* function. **Conclusions**: These findings emphasize the critical role of *SREBF2* in regulating both triglyceride and cholesterol biosynthesis, providing valuable insights into its functions in buffalo mammary glands.

## 1. Introduction

Sterol regulatory element-binding transcription factor 2 (SREBF2), also known as sterol regulatory element-binding protein 2 (SREBP2) or basic helix-loop-helix protein 2 (bHLHd2), was first cloned from human HeLa cells in 1993 [1]. SREBF2 is a sterol regulatory element-binding transcription factor family member and functions as a basic helix-loop-helix leucine zipper (bHLH-Zip) transcription factor [2]. It primarily regulates the expression of genes involved in cholesterol biosynthesis and uptake, with its activity being tightly controlled through a negative feedback mechanism [3]. When intracellular cholesterol levels are low, SREBF2, in conjunction with its escort protein sterol regulatory element-binding protein cleavage-activating protein (SCAP), is transported from the endoplasmic reticulum (ER) to the Golgi apparatus via coat protein complex II (COPII) vesicles. In the Golgi, SREBF2 undergoes cleavage and activation, then translocates to the nucleus to regulate gene expression [4]. Conversely, when intracellular cholesterol levels rise, SREBF2 and SCAP interact with the insulin-induced gene (*INSIG*) encodes protein, forming the SREBF-SCAP-INSIG complex, which is retained in the ER [5]. This interaction prevents SREBF2 from regulating genes involved in cholesterol synthesis [6].

In tissues with active lipid metabolism, SREBF2 primarily regulates the expression of genes involved in cholesterol synthesis. However, in *SREBF2* knockdown (*SREBF2*^−/−^) mouse embryos, the expression of genes involved in fatty acid biosynthesis was reduced [7]. Another study showed that the knockdown of *SREBF2* in the mouse liver led to a decrease in the expression of genes related to both cholesterol biosynthesis and uptake, as well as fatty acid synthesis [8]. These finding indicates that SREBF2 regulates cholesterol synthesis and plays a significant role in fatty acid synthesis within the liver [8]. In goats, the phosphoinositide 3-kinase (PI3K)/protein kinase B (AKT)/mammalian target of rapamycin (mTOR) pathway is involved in milk fat synthesis within the goat mammary epithelial cells (GMECs) [9]. Nonetheless, the molecular mechanisms by which SREBF2 regulates lipid metabolism, especially in buffalo mammary glands, remain unclear.

Domestic buffalo are classified into swamp buffalo (2n = 48) and river buffalo (2n = 50), and they are primarily found in Asia [10]. Swamp buffalo are known for their docile nature and endurance and are mainly used as draft animals. In contrast, river buffalo are larger and have higher milk production, making them a primary source of dairy [11]. Due to the nutritional benefits of buffalo milk, it has become the second-largest milk source worldwide [12]. Compared to Holstein cow milk, buffalo milk has higher levels of fat, protein, and total solids [13], containing up to 7.9% fat, 4.5% protein, and 18.4% total solids [14]. Consequently, the function and molecular mechanisms of genes involved in buffalo lactation have become a research focus. Although the role of the *SREBF2* gene has been studied in some other species, its function in buffalo, especially in its mammary lactation process, remains poorly understood. Therefore, in this study, we cloned the *SREBF2* gene CDS from the mammary gland tissue of the Binglangjiang buffalo. We then compared this sequence with homologous sequences from other species and performed a detailed analysis of the gene structure and the encoded protein’s physicochemical properties, structure, and function. Additionally, we examined the expression of this gene in multiple buffalo tissues and performed cellular-level functional experiments to assess changes in the expression of genes related to fatty acid and cholesterol synthesis, as well as variations in triglyceride and cholesterol content. Finally, we investigated the population variation in the *SREBF2* CDS in river and swamp buffalo. This study will provide new insights into the role of *SREBF2* in buffalo triglyceride and cholesterol biosynthesis, along with its regulatory mechanisms.

## 2. Materials and Methods

### 2.1. Sample Collection

Under identical feeding and management conditions, the tissue samples from six healthy female Binglangjiang buffalo (river type) were selected and collected in Tengchong, Yunnan Province, China. Three buffalo were in lactation (5 years old, approximately 60 days postpartum), while the other three were in the dry period (5 years old, approximately 60 days before parturition). Following slaughter, tissue samples from the heart, liver, spleen, lungs, kidneys, pituitary gland, brain, mammary gland, duodenum, rumen, and muscle were promptly collected, transferred to RNase-free cryovials, snap-frozen in liquid nitrogen, and stored at −80 °C for subsequent total RNA extraction.

To detect single nucleotide polymorphisms (SNPs) in the *SREBF2* CDS, blood samples were collected from 65 adult Binglangjiang buffalo (river type) and 65 adult Dehong buffalo (swamp type), with no direct kin relationship between individuals. The samples were taken from their core distribution areas in Tengchong and Mangshi, Yunnan Province, China, respectively. During the sampling process, in order to ensure that the sampled individuals had no direct blood relationships, thereby ensuring the representativeness of the samples and the reliability of the data, we first conducted interviews with buffalo farmers or farm owners and carefully reviewed the archival materials provided by them. Based on this, this study only collected blood samples from one or two buffalo in each village or farm. Approximately 4 mL of blood were collected from each buffalo, placed into a centrifuge tube containing EDTA anticoagulant, and then stored at −20 °C for DNA extraction.

### 2.2. Isolation and Identification of the SREBF2 Gene

Total RNA was extracted from tissue samples according to the manufacturer’s instructions for RNAiso Plus reagent (TaKaRa, Dalian, China). The RNA concentration and purity were measured with a NanoDrop 2000 UV-Vis spectrophotometer (Thermo Fisher Scientific, Waltham, MA, USA), and integrity was assessed via 1% agarose gel electrophoresis. cDNA was synthesized from 2 to 3 μg of RNA using the M-MLV Reverse Transcriptase (RNase H^-^) reagent (TaKaRa, Dalian, China). The resulting cDNA was diluted to a concentration of 100 ng/μL and stored at −20 °C.

Primers for isolating the *SREBF2* CDS from buffalo were designed using Primer Premier 5 [15] (Table S1). The *SREBF2* CDS was amplified using cDNA from buffalo mammary gland tissue as a template. The 20 μL PCR reaction mixture contained 2 μL of 10× LA PCR Buffer II (Mg^2+^ Plus), 3.2 μL of dNTPs (each 2.5 mmol/L), 0.4 μL of each upstream and downstream primer (10 μM), 0.1 μL of LA Taq enzyme (5 U/μL, TaKaRa, Dalian, China), 2 μL of cDNA template (100 ng/μL), and 11.9 μL of ddH_2_O. The PCR program included an initial denaturation at 95 °C for 3 min, followed by 35 cycles of 94 °C for 30 s, annealing for 40 s (annealing temperature provided in Table S1), and at 72 °C for 2 min. A final extension was performed at 72 °C for 5 min, and the reaction terminated at 4 °C. PCR products were analyzed by 1% agarose gel electrophoresis, and the target band was purified using the TaKaRa MiniBEST Agarose Gel DNA Extraction Kit Ver.4.0 (TaKaRa, Dalian, China) following the manufacturer’s protocol. The purified PCR product was ligated into the pMD18-T vector (TaKaRa, Dalian, China) and cloned. Twenty clones were selected for bidirectional sequencing of the target fragment.

The *SREBF2* gene sequence obtained from buffalo was proofread and analyzed using SeqMan from Lasergene (DNASTAR Inc., Madison, WI, USA). Open reading frames (ORFs) were identified using the ORF Finder tool (https://www.ncbi.nlm.nih.gov/orffinder, accessed on 17 November 2024). To confirm the identity of the sequence, a homologous search was performed using the online BLAST program (https://blast.ncbi.nlm.nih.gov/Blast.cgi, accessed on 17 November 2024) in the NCBI database.

### 2.3. Gene Sequence and Structural Analysis

To investigate the sequence features of *SREBF2* in buffalo, FASTA and GTF/GFF files for 13 species, including those related to buffalo, were retrieved from the NCBI database. The *SREBF2* gene and its encoded protein sequences were downloaded for a comparative analysis. A chromosome map of buffalo was generated using the Gene Location Visualize from GTF/GFF function in TBtools software v2.154 [16]. Codon usage bias was assessed with the codonW [17]. The GXF Fix function in TBtools was utilized to incorporate mRNA and UTR information for *SREBF2* from each species. The relative positions of the Exons, CDS, and untranslated regions within each transcript were calculated in Excel. The processed data were compiled into a Browser Extensible Data (BED) file format and visualized using the Gene Structure Display Server 2.0 [18].

### 2.4. Physicochemical Properties, Motifs, and Structure

The physicochemical properties of SREBF2 were predicted using ProtParam [19]. Transmembrane domains, signal peptides, and functional modification sites were analyzed with TMHMM 2.0 [20], SignalP 5.0 [21], and Prosite [22], respectively. The consistency of the SREBF2 amino acid sequence across 13 species was evaluated using the MegAlign program in Lasergene v7.1 (DNASTAR Inc., Madison, WI, USA). A phylogenetic tree of SREBF2 across species was constructed using the Maximum Likelihood method (JTT+G model) in Mega7 [23] with 5000 bootstrap replications. A motif analysis of SREBF2 amino acid sequences from various species was conducted using the MEME tool [24], while a conserved domain analysis was performed using the NCBI Batch Web CD-Search tool [25]. The results from the phylogenetic, motif composition, and conserved domain analyses were integrated and visualized with the Gene Structure View (Advanced) tool in TBtools.

The subcellular localization, secondary structure, and 3D structure of SREBF2 were predicted using WoLF PSORT [26], SOPMA [27], and SWISS-MODEL [28], respectively. The protein–protein interaction network of SREBF2 was predicted using STRING [29], and functional annotation was performed with InterProScan [30] and DAVID [31]. Synteny plots of *SREBF2* in buffalo and other Bovidae species were generated using the One Step MCScanX, Advanced Circos, and Multiple Synteny Plot functions in TBtools.

### 2.5. Differential Expression Analysis in Multiple Tissues

RT-qPCR was conducted to evaluate the tissue-specific expression of *SREBF2* in 11 tissues of Binglangjiang buffalo. β-actin (*ACTB*) was used as the reference gene. The analysis was performed following the manufacturer’s protocol for the SYBR Premix Ex Taq reagent (TaKaRa, Dalian, China) on a QuantGene 9600 instrument (Bioer, Hangzhou, China). Each experiment was repeated three times to ensure reliability. The RT-qPCR primers used are listed in Table S1. The total reaction volume was 20 μL, consisting of 10 μL SYBR Premix Ex Taq, 0.8 μL of upstream and downstream primers (10 μmol L^−1^), 6.4 μL of ddH_2_O, and 2 μL of cDNA template (100 ng μL^−1^). The reaction conditions were carried out according to the manufacturer’s guidelines.

In order to further analyze the expression of *SREBF2* in buffalo mammary glands during early (30–54 days postpartum), middle (117–136 days postpartum), and late (250–273 days postpartum) lactation, we downloaded the raw data of buffalo mammary tissue RNA sequencing (RNA-seq) from the NCBI database with the BioProject ID PRJNA453843 [32]. The data include four animals in each group during early, middle, and late lactation. All the selected animals were in their third parity, aged between 6.5 and 7 years, and had a milk yield ranging from 7 to 8 L per day [32]. After obtaining the raw data, quality control was first performed using FastQC (http://www.bioinformatics.babraham.ac.uk/projects/fastqc, accessed on 17 November 2024), followed by the removal of adapter sequences and low-quality data from the raw files using Trimmomatic [33]. Then, we used FastQC again to re-evaluate the data quality. Finally, kallisto was used to calculate transcripts per kilobase million (TPM) as the expression abundance of *SREBF2* [34]. The average TPM values for each period were calculated separately and visualized using GraphPad Prism 9.5 software (GraphPad Software Inc., La Jolla, CA, USA).

### 2.6. Cell Culture

In this study, we used mammary epithelial cells (BuMECs) that were previously isolated, purified, and identified by our group to conduct cell-level experiments [35]. The picture of BuMECs taken with a TS100 microscope (Nikon, Shanghai, China) is shown in Figure S1. The BuMECs were removed from liquid nitrogen and cultured in DMEM/F12 medium (Gibco, Carlsbad, CA, USA) after resuscitation; the medium was supplemented with 10% fetal Bovidae serum (Gibco, Carlsbad, CA, USA), 10 kU L^−1^ penicillin/streptomycin (Gibco, Carlsbad, CA, USA), 5 μg mL^−1^ hydrocortisone (Sigma, St. Louis, MO, USA), 5 μg mL^−1^ insulin (Sigma, St. Louis, MO, USA), and 1 μg mL^−1^ epidermal growth factor (Sigma, St. Louis, MO, USA), and it was cultured at 37 °C with 5% CO_2_. Forty-eight hours before the experiment, 3 μg mL^−1^ prolactin (Sigma, St. Louis, MO, USA) was added to the medium to induce lactation in BuMECs [36]; the medium was then replaced with DMEM/F12 containing 10% fetal bovine serum with no hormones or growth factors [37].

### 2.7. siRNA Transfection

Two pairs of specific small interfering RNAs (siRNAs) targeting different regions of the *SREBF2* CDS (siRNA1-*SREBF2* and siRNA2-*SREBF2*) and a non-specific negative control siRNA (siRNA-NC) were designed and synthesized by Shanghai Shenggong Biotechnology Co., Ltd. (sequences provided in Table S1). When BuMECs reached 70–80% confluence, siRNA1-*SREBF2*, siRNA2-*SREBF2*, and siRNA-NC (100 nM each) were transfected into the cells using Trans Lipid Transfection Reagent (TransGen BioTech, Beijing, China) in accordance with the manufacturer’s protocol. The interference efficiency of the siRNAs was assessed by measuring *SREBF2* mRNA levels using qPCR. The siRNA showing the highest interference efficiency was selected for subsequent experiments. After 48 h of transfection, BuMECs were collected for RT-qPCR analysis, and all assays were performed in triplicate.

### 2.8. Analysis of Triglyceride (TAG) and Total Cholesterol (T-CHO) Levels in Cells

After 48 h of *SREBF2* knockdown in BuMECs, the cells were washed twice with PBS. Intracellular TAG levels were measured using a Triglyceride Assay Kit (GPO-POD, Applygen Technologies Inc., Beijing, China) following the manufacturer’s instructions. Total protein concentration was determined using a BCA Protein Assay Kit (Beyotime Biotechnology Co., Ltd., Shanghai, China). TAG content was normalized to protein levels (per mg protein).

The total cholesterol (T-CHO) concentration in BuMECs was measured using a Total Cholesterol Assay Kit (COD-PAP, Nanjing Jiancheng Bioengineering Research Institute, Nanjing, China). Cholesterol content was calculated using the following formula: cholesterol content (mmol/gprot) = ([A_sample_ − A_blank_]/[A_standard_ − A_blank_]) × C_standard_/C_pr_, where C_standard_ (mmol/L) is the standard concentration and C_pr_ (gprot/L) is the protein concentration in the homogenate.

### 2.9. Polymorphism Detection and Analysis

Genomic DNA was extracted from blood samples using the phenol–chloroform method [38]. SNP detection primers were designed based on the *SREBF2* sequence of buffalo (NC_059160; Table S1). The PCR reaction mixture (20 μL) consisted 2 µL of 10× PCR Buffer (Mg^2+^ Plus), 1.6 µL of dNTPs (2.5 mmol/L), 0.4 µL of each primer (10 µM), 0.1 µL of rTaq enzyme (5 U/μL), 2.0 µL of DNA template (100 ng/μL), and 13.5 µL of ddH_2_O. The PCR program included an initial denaturation at 95 °C for 3 min; 35 cycles of 94 °C for 30 s; annealing for 30 s (annealing temperatures provided in Table S1); and 72 °C for 20–30 s; followed by a final extension at 72 °C for 5 min, after which the reaction was terminated. PCR products were analyzed via agarose gel electrophoresis and bidirectionally sequenced by Shanghai Biotechnology Co., Ltd. (Shanghai, China).

Sequences were checked and aligned using SeqMan software (DNASTAR Inc., Madison, WI, USA). The relative synonymous codon usage rate (RSCU) of the CDS was analyzed with CodonW v1.4.2 (http://codonw.sourceforge.net/, accessed on 17 November 2024). Multiple sequences were aligned using Clustal X [39], followed by manual adjustment with BioEdit [40], and then nucleotide and amino acid variation sites were output using MEGA7 software [23]. Genotype and allele frequencies were calculated, and the Hardy–Weinberg equilibrium was assessed using PopGen32 software [41]. Haplotypes are defined using PHASE software v2.0 [42]. The PANTHER program (http://www.pantherdb.org/, accessed on 17 November 2024) was used to predict the effects of amino acid substitutions on protein function.

### 2.10. Data Analysis

RT-qPCR data were analyzed using the 2^−ΔΔCt^ method. Results from three independent replicates are presented as the means ± standard error of the mean (SEM). Statistical analysis and visualization were performed using GraphPad Prism 9.5 software (GraphPad Software Inc., La Jolla, CA, USA). Differences between the two groups were assessed using a Student’s *t*-test, while multiple group comparisons were analyzed using a one-way analysis of variance (ANOVA) followed by a Tukey’s test. Statistical significance was set at *p* < 0.05.

## 3. Results

### 3.1. Cloning and Identification of the Buffalo SREBF2 Gene

Using complementary DNA (cDNA) from lactating buffalo mammary tissue as a template, the CDS of the *SREBF2* gene was successfully cloned (Figure S2). The open reading frame (ORF) was identified using ORF Finder, and a homology search was conducted using BLAST from the NCBI database. The results revealed that the CDS shares strong correspondence (>96%) with the *SREBF2* CDSs from other bovine species. Specifically, the similarity with buffalo (XM_025282779.2), cattle (NM_001205600.2), zebu (XM_019961897.1), bison (XM_010843698.1), yak (XM_014481975.1), goat (XM_018048827.1), and sheep (XM_027968156.2) was 99.79%, 97.84%, 97.54%, 97.56%, 98.03%, 96.66%, and 96.54%, respectively. The *SREBF2* CDS has been submitted to the NCBI database under accession number KU508423.1. This sequence is 3327 base pairs (bps) long and encodes a protein comprising 1108 amino acid residues (Figure S3) with the protein accession number AOT28252.1. The nucleotide composition of the *SREBF2* CDS is as follows: A = 18.58%, G = 30.24%, T = 16.89%, C = 34.30%, with a C+G content of 64.54%.

### 3.2. Comparison of the Transcriptional Region Structure of SREBF2 Gene

**Figure 1:**
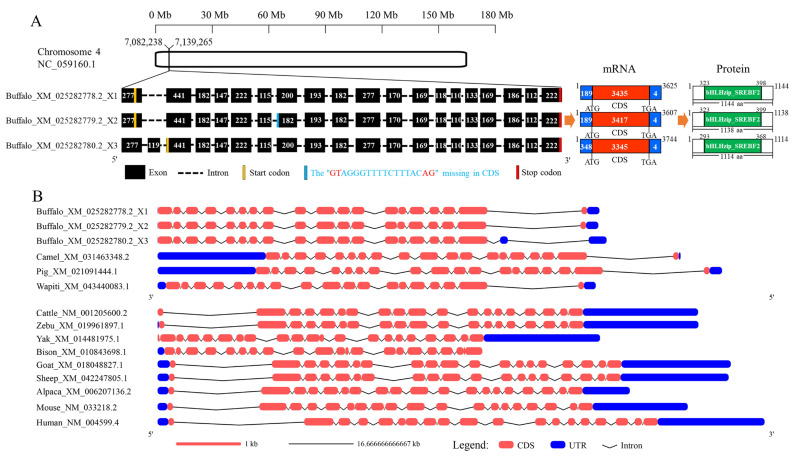
Transcriptional region structure of the *SREBF2* gene. (**A**) Comparison of the transcriptional region structures of buffalo *SREBF2* transcript variants. (**B**) Comparison of the transcriptional region structure of buffalo *SREBF2* with those of other mammalian species.

The *SREBF2* gene is located on buffalo chromosome 4 (NC_059160.1), and there are three transcripts in the NCBI database (https://www.ncbi.nlm.nih.gov/gene, accessed on 17 November 2024): XM_025282778.2_X1, XM_025282779.2_X2, and XM_025282780.2_X3 (Figure 1A). Both XM_025282778.2_X1 and XM_025282779.2_X2 consist of 19 exons and 18 introns, while XM_025282780.2_X3 includes 20 exons and 19 introns. Compared to XM_025282778.2_X1, exon 7 of XM_025282779.2_X2 is missing 18 bp, in accordance with the GT-AG splicing rule. Notably, the *SREBF2* sequence obtained in this study also lacks this 18 bp, indicating that it closely resembles XM_025282779.2_X2. Further comparisons revealed that buffalo and other bovine species differ in the structure of the transcribed region of the *SREBF2* gene, mainly in the untranslated regions (UTRs) and the intronic regions. It is noteworthy that the CDS structures of XM_025282778.2_X1 and XM_025282779.2_X2 are more similar to those of other bovid species than XM_025282780.2_X3 (Figure 1B).

### 3.3. Sequence Identity, Motif Composition, Structure, and Evolutionary Relationships

**Figure 2:**
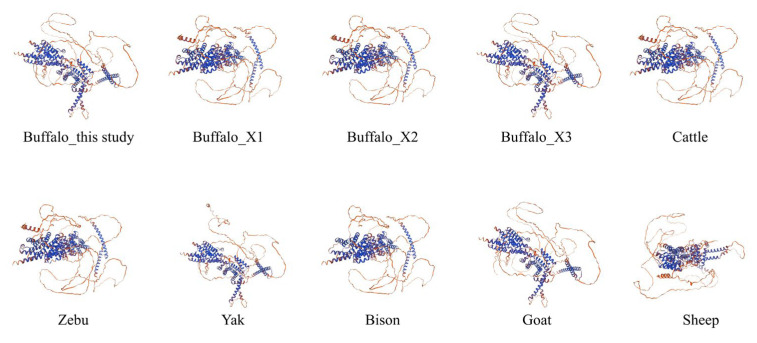
Three-dimensional structures of the SREBF2 protein in buffalo and other Bovidae species. Predicted local similarity to target is shown in different colors, shown in blue above average model confidence (pLDDT, %) and orange below pLDDT.

**Figure 3:**
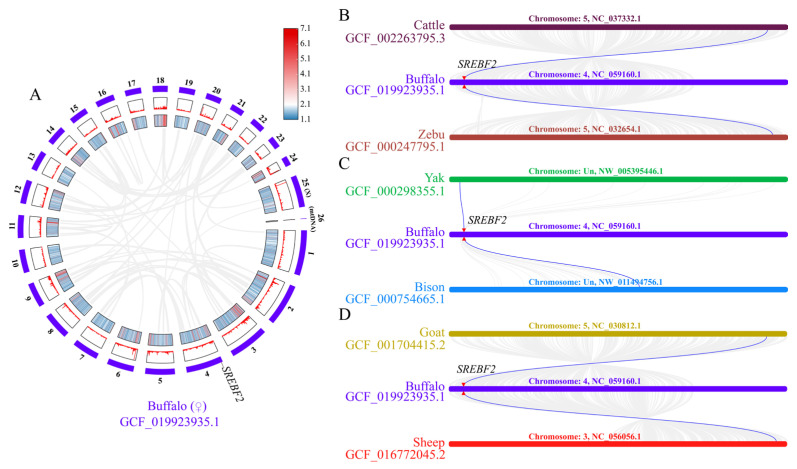
A collinearity analysis of the *SREBF2* gene. (**A**) Collinearity analysis of *SREBF2* in river buffalo, with chromosomes 1 to 24 marked (25 and 26 represent the X chromosome and mitochondrial DNA, respectively); gene density is represented by heat maps and line plots. (**B**) The collinearity of *SREBF2* in buffalo, cattle, and zebu. (**C**) Collinearity of *SREBF2* in buffalo, yak, and bison. (**D**) Collinearity of *SREBF2* in buffalo, goat, and sheep.

To examine the similarity of the buffalo SREBF2 amino acid sequence with that of other species, an alignment analysis was performed using sequences obtained from the NCBI database (Figure S4). The results showed that the buffalo SREBF2 sequence exhibited strong correspondence to the sequences from 12 other species, exceeding 94% similarity with other Bovidae species and over 91% with other mammalian species. To further assess the sequence characterization of the SREBF2, a phylogenetic tree was constructed based on amino acid sequences from the 13 mammalian species, followed by an analysis of motif composition and conserved structures. A phylogenetic analysis showed that buffalo and other bovids clustered into a large branch, indicating that SREBF2 has little sequence difference and similar functions across bovine species (Figure S5A). A motif and domain composition analysis revealed that the domain compositions of SREBF2 in buffalo and other bovids all contain the bHLHzip-SREBP2 domain (Figure S5B), which plays a key role in regulating lipid and cholesterol biosynthesis (Figure S5C). Notably, the bHLHzip-SREBP2 domain includes motif 1 and motif 7 (Figure S5). Secondary structure analysis revealed that the buffalo SREBF2 protein comprises 42.60% α-helix, 2.8% extended chain, 0.99% β-turn, and 53.61% random coil (Table S2). Homology modeling based on the buffalo SREBF2 amino acid sequence demonstrated 92.42% similarity to the template (Q12772.1.A of *Homo sapiens*). Further analyses confirmed that the 3D structure of SREBF2 in bovine species exhibits a high degree of similarity (Figure 2) to buffalo, cattle, yak, bison, and goat, with over 90% identity to the template Q12772.1.A. Interestingly, although no collinearity of SREBF2 was observed within the buffalo genome (Figure 3A), it was present in cattle, zebu, yak, bison, goat, sheep (Figure 3B–D). This finding further highlights the conserved functional roles of SREBF2 in Bovidae species.

### 3.4. Properties and Functional Modifications of SREBF2 Protein

The theoretical isoelectric point (pI) of buffalo SREBF2 is 9.04, with an instability index of 53.89, an aliphatic index of 89.49, and an average hydrophobicity of −0.12. These values suggest that the protein is unstable and has low hydrophilicity. The absence of a signal peptide indicates that buffalo SREBF2 is a non-secretory protein. Further predictions reveal that buffalo SREBF2 possesses a transmembrane structure and is localized to the nucleus (score: 10), plasma membrane (score: 12), and cytosol (score: 8), respectively. Interestingly, the average hydrophobicity of −0.12 is consistent across SREBF2 proteins from buffalo, cattle, bison, and sheep. Overall, the physicochemical properties of SREBF2 are highly similar across Bovidae species (Table S3). Functional modification site predictions also show that buffalo SREBF2 shares six kinds of functional modification sites with other Bovidae species (Figure S6).

### 3.5. Protein Interactions, Gene Ontology Analysis

The results from STRING prediction identified the main proteins interacting with buffalo SREBF2 (Figure S7). These proteins are classified into four groups: The first group primarily involves fatty acid synthesis, including the fatty acid synthase (FASN) and sterol regulatory element-binding transcription factor 1 (SREBF1) protein. The second group is primarily related to cholesterol synthesis, including the niemann-pick c1 (NPC1), 3-hydroxy-3-methylglutaryl-coenzyme a reductase (HMGCR), and NPC1-like intracellular cholesterol transporter 1 (NPC1L1) proteins. The third group consists of transcriptional regulators, including membrane-bound transcription factor site-2 protease (MBTPS2), SREBP cleavage-activating protein (SCAP), insulin-induced gene 1 (INSIG1), and insulin-induced gene 2 (INSIG2), and SREBF1. The fourth group involves proteins associated with nuclear transport, including karyopherin subunit beta 1 (KPNB1).

The molecular function analysis suggests that buffalo SREBF2 is involved in protein dimerization activity (GO:0046983), RNA polymerase II cis-regulatory region sequence-specific DNA binding (GO:0000978), and DNA-binding transcription factor activity that is RNA polymerase II-specific (GO:0000981). In terms of biological processes, SREBF2 plays roles in the positive regulation of cholesterol storage (GO:0010886), the positive regulation of transcription by RNA polymerase II (GO:0045944), and the regulation of transcription by RNA polymerase II (GO:0006357). Regarding cellular components, SREBF2 acts primarily in the nucleus (GO:0005634). These findings suggest that SREBF2 likely plays a pivotal role in regulating fatty acid and cholesterol synthesis in buffalo.

### 3.6. Multi-Tissue Expression Analysis of SREBF2 in Buffalo

**Figure 4:**
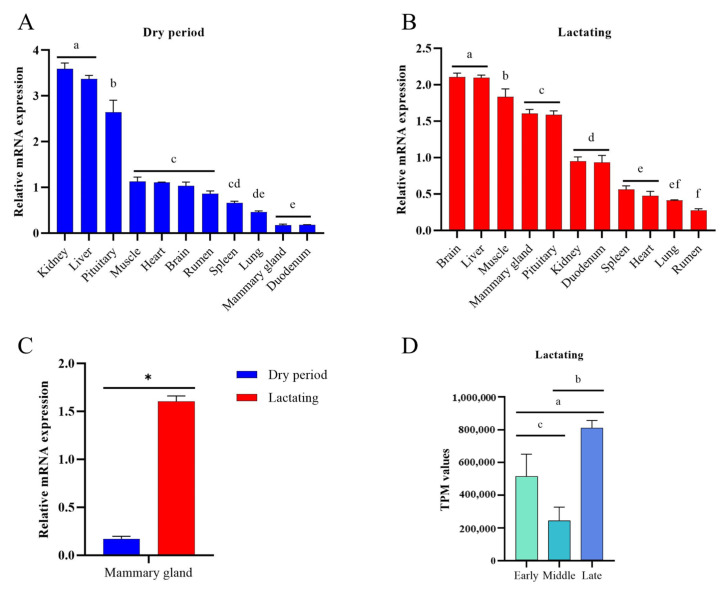
The expression profile of *SREBF2* in multiple tissues of buffalo. (**A**) Differential expression of the *SREBF2* gene in 11 tissues of buffalo during the dry period. (**B**) Differential expression of the *SREBF2* gene in 11 tissues of buffalo during lactation. (**C**) Differential expression of the *SREBF2* gene in the mammary gland of buffalo during dry and lactating periods. (**D**) Expression of *SREBF2* in the mammary gland of buffalo during early, middle, and late lactation. Different letters (a–f) indicate significant differences between groups (*p* < 0.05), and “*” also indicates significant differences (*p* < 0.05).

To evaluate the expression of *SREBF2* in buffalo tissues involved in lipid metabolism, multi-tissue expression profiles were constructed for both the dry and lactation periods. The mRNA expression levels of *SREBF2* in 11 different buffalo tissues during the dry and lactation periods are shown in Figure 4A,B, respectively. *SREBF2* was detected in all tissues during both periods. During the dry period, *SREBF2* expression was highest in the kidney and liver, followed by the pituitary, muscle, heart, brain, and rumen, with lower expression observed in the mammary gland and duodenum. In contrast, the highest *SREBF2* mRNA levels were detected in the brain and liver, followed by the skeletal muscles, mammary gland, and pituitary gland, while the rumen exhibited the lowest expression. A further analysis revealed that *SREBF2* mRNA levels in the mammary gland were significantly higher during lactation compared to the dry period (Figure 4C), suggesting a functional role for *SREBF2* in the mammary gland during lactation. Based on transcriptome data from 12 lactating buffalo mammary glands, a further analysis showed that the expression level of *SREBF2* was significantly increased in late lactation compared with middle lactation (*p* < 0.05; Figure 4D).

### 3.7. Functional Assays at the Cellular Level

**Figure 5:**
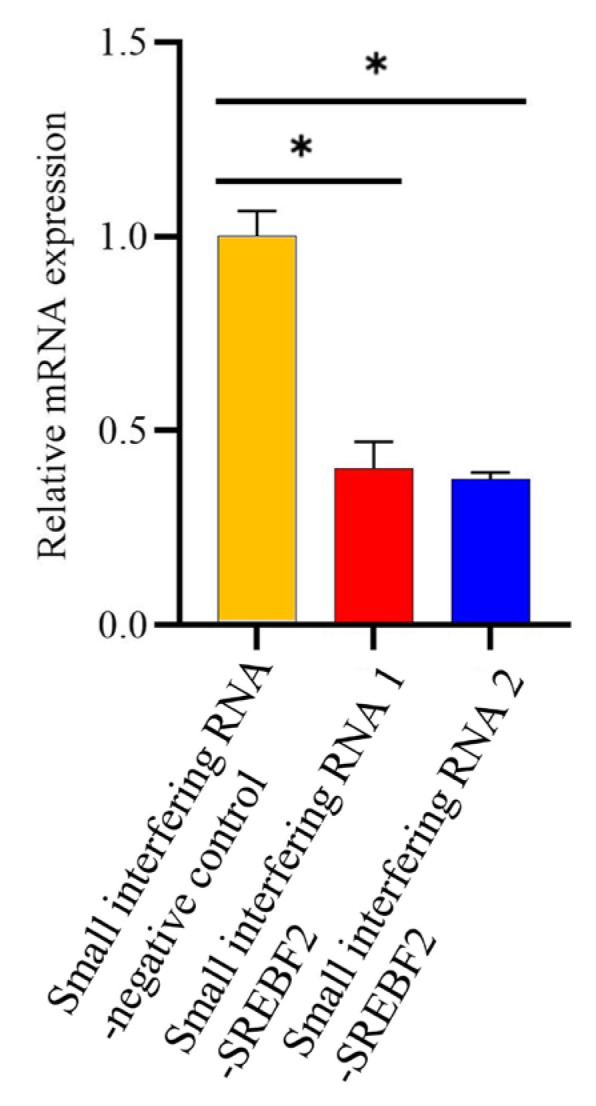
Quantitative real-time polymerase chain reaction analysis of interference efficiency of the two specific small interfering RNAs on *SREBF2* in prolactin-induced buffalo mammary epithelial cells. “*” indicates significant differences (*p* < 0.05).

To investigate the role of *SREBF2* in buffalo triglyceride and cholesterol biosynthesis, functional assays were performed using BuMECs. Initially, BuMECs (induced with prolactin) were transfected with siRNA1-*SREBF2* and siRNA2-*SREBF2* to determine the siRNA with the highest interference efficiency. The results (Figure 5) showed that, compared to the control group, the *SREBF2* mRNA levels decreased by 59.78% (*p* < 0.05) and 62.48% (*p* < 0.05) following transfection with siRNA1-*SREBF2* and siRNA2-*SREBF2*, respectively. These findings indicate that siRNA2-*SREBF2* achieved the highest interference efficiency and was selected for subsequent experiments.

**Figure 6:**
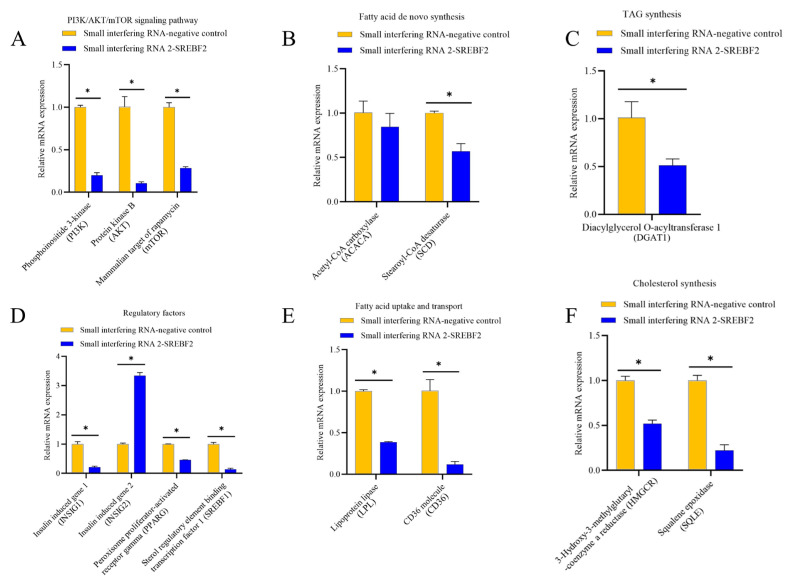
Comparison of the expression of genes involved in triglyceride and cholesterol synthesis following *SREBF2* knockdown. (**A**) Expression levels of genes in the *PI3K*/*AKT*/*mTOR* signaling pathway. (**B**) Expression levels of genes involved in de novo fatty acid synthesis. (**C**) Expression levels of genes take part in triglyceride (TAG) synthesis. (**D**) Expression levels of genes that code for regulatory factors. (**E**) Expression levels of genes involved in fatty acid uptake and transport. (**F**) The expression levels of genes participating in cholesterol synthesis. “*” indicates significant differences (*p* < 0.05).

**Figure 7:**
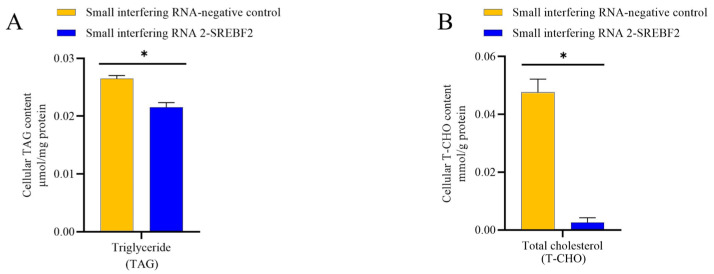
Decreased levels of TAG (**A**) and total cholesterol (**B**) in buffalo mammary epithelial cells following *SREBF2* knockdown. “*” indicates significant differences (*p* < 0.05).

To explore the role of the *SREBF2* gene in buffalo mammary gland triglyceride and cholesterol biosynthesis, we evaluated the expression of key genes involved in lipid metabolism after *SREBF2* knockdown in BuMECs (Figure 6). These genes include those associated with lipid synthesis pathways (*PI3K*, *AKT*, *mTOR*), regulatory factors (*INSIG1*, *INSIG2*, Peroxisome proliferator-activated receptor gamma [*PPARG*], *SREBF1*), de novo fatty acid synthesis (Acetyl-CoA carboxylase [*ACACA*], Stearoyl-CoA desaturase [*SCD*]), fatty acid uptake and transport (Lipoprotein lipase [*LPL*], CD36 molecule [*CD36*]), TAG synthesis (Diacylglycerol O-acyltransferase 1 [*DGAT1*]), and cholesterol synthesis (*HMGCR*, Squalene epoxidase [*SQLE*]). Following *SREBF2* knockdown, the expression of *PI3K*, *AKT*, and *mTOR* was significantly reduced by 80.08% (*p* < 0.05), 89.25% (*p* < 0.05), and 71.79% (*p* < 0.05), respectively. Regulatory factors *INSIG1*, *PPARG*, and *SREBF1* were significantly downregulated by 78.97% (*p* < 0.05), 54.52% (*p* < 0.05), and 86.68% (*p* < 0.05), respectively, while *INSIG2* expression was upregulated three-fold (*p* < 0.05). The fatty acid de novo synthesis gene *SCD* was significantly downregulated by 43.1% (*p* < 0.05), while no change was observed in *ACACA* expression. Fatty acid uptake and transport genes, *LPL* and *CD36*, were significantly decreased by 61.40% (*p* < 0.05) and 88.30% (*p* < 0.05), respectively. The TAG synthesis gene *DGAT1* was downregulated by 49.32% (*p* < 0.05), while cholesterol synthesis genes *HMGCR* and *SQLE* were significantly decreased by 48.06% (*p* < 0.05) and 77.95% (*p* < 0.05), respectively. In addition, *SREBF2* knockdown resulted in a reduction in TAG and cholesterol content in BuMECs by 23.53% (*p* < 0.05) and 94.56% (*p* < 0.05), respectively (Figure 7).

### 3.8. Sequence Variations and Population Genetic Analysis

**Figure 8:**
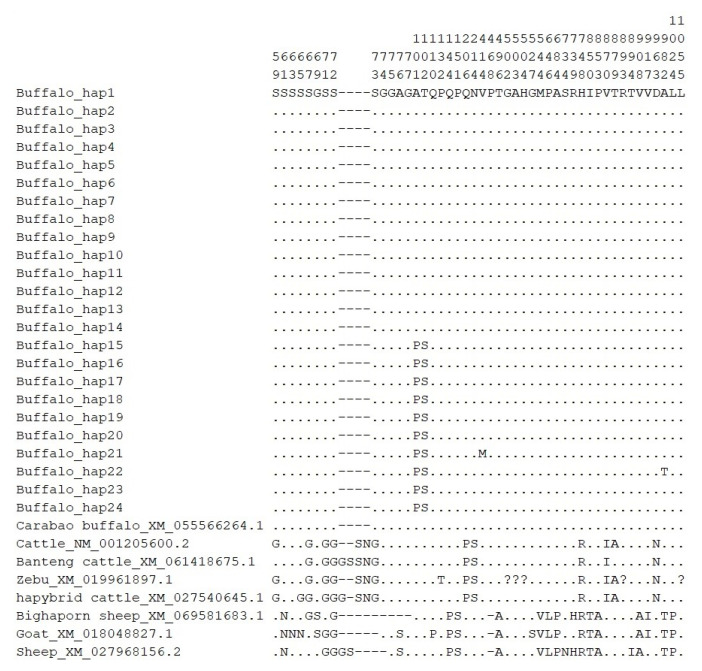
Amino acid sequence differences in SREBF2 between buffalo and other Bovidae species. The numbers correspond to the positions of amino acids in the mature peptide. A dot (.) denotes identity with the SREBF2 sequence, and amino acid substitutions are represented by different letters. Missing data are marked with a question mark (?). Horizontal line (-) represents a deletion in the sequences.

In this study, a total of 22 SNPs were identified in the *SREBF2* CDS of two types of buffalo. The results showed that substitutions c.477G>A, c.636C>G, c.795G>A, c.1095T>C, c.2017C>T, c.2079C>T and c.2475A>G were only found in river buffalo, whereas c.246C>T, c.843C>T, c.1240G>A, c.2505C>T, and c.2944G>A were unique to swamp buffalo. The substitutions c.2082T>A, c.2799C>T, c.3186C>T and c.3354G>A were shared by both types of buffalo (Table 1). It is worth noting that c.252, c.300, c.301, and c.304 have been purified. In river buffalo, the corresponding haplotypes are CC, GG, GG, and AA, respectively. In contrast, the haplotypes in swamp buffalo are TT, TT, CC, and TT for these SNPs. The substitutions found in river buffalo were all synonymous, while, in swamp buffalo, four non-synonymous substitutions were identified: c.301G>C, c.304A>T, c.1240G>A, and c.2944G>A, resulting in the amino acid changes p.101A>P, p.102T>S, p.414V>M, and p.982A>T (Figure 8), respectively. The evaluation showed that they had no effect on the functions of *SREBF2*. Meanwhile, a synonymous substitution (c.2799C>T) was found only in swamp buffalo, which is the CC homozygote in river buffalo. Further analysis showed that the RSCU values of buffalo *SREBF2* did not differ much, suggesting that the synonymous substitutions found here were unlikely to have a substantial impact on the function of *SREBF2* by changing codon usage frequency (Table S4).

Based on SNPs identified in buffalo *SREBF2* CDS, a total of 24 haplotypes were defined, named Buffalo_hap1 to Buffalo_hap24 (Table S5). Among them, Buffalo_hap1 is the dominant haplotype, with a frequency of 0.197559. Buffalo_hap20 has the lowest haplotype frequency, 0.010202 (Table S5). Buffalo_hap1 to Buffalo_hap14 are found in river buffalo, while Buffalo_hap15 to Buffalo_hap24 are found in swamp buffalo.

To investigate sequence differences, we compared the buffalo haplotype sequences with homologous sequences of Bovidae species downloaded from the NCBI. The results showed that nucleotide substitution c.252C>T, c.342C>G, and c.477G>A were found only in *Bubalus* (Figure 8, Figure S8). The sites c.617 (p.206), c.641 (p.214), c.2608 (p.870), and c.2887 (p.963) in *SREBF2* distinguish the *Bubalus* and *Ovis* from the *Bos*.

## 4. Discussion

Buffalo milk is increasingly favored for its rich nutrient content and high nutritional and biological activity, especially in regard to its potential benefits for specific groups such as growing children, lactating mothers, high-intensity workers, and the elderly [43]. Understanding the synthesis and regulation of key components like fat and cholesterol in buffalo milk is essential for developing healthier and more appealing buffalo dairy products [44]. In dairy cows, SREBF2 plays a pivotal role in milk fat metabolism by coordinating transcription factors like SREBF1 and PPARG to enhance the expression of genes involved in fatty acid synthesis, including *ACACA* and *SCD* [45]. Moreover, studies have shown that, during lactation, the expression of *SREBF2* in the liver increases, directly regulating the expression of cholesterol biosynthesis-related genes, such as *HMGCR* and *SQLE* [46]. However, the molecular mechanisms by which SREBF2 regulates milk fat synthesis in buffalo remain unclear and warrant further investigation.

*SREBF2* CDS has also been cloned in humans [1], pigs [47], and chickens [48]. In this study, we successfully cloned and characterized the CDS of the *SREBF2* gene from buffalo mammary glands. The sequence exhibited over 96% identity with those of other Bovidae species. Our findings demonstrated that buffalo SREBF2 closely resembles other Bovidae species in physicochemical properties, motif composition, conserved domains, and more advanced structural features, including secondary and tertiary structures. Furthermore, phylogenetic and collinearity analysis findings suggest that buffalo *SREBF2* shares conserved functional roles with *SREBF2* in other Bovidae species.

The tissue expression of *SREBF2* has been studied extensively across various species in recent years, including pigs (in liver, muscle, and adipose tissue) [49], rabbits (in liver and adipose tissue) [50], chickens (in the heart, liver, spleen, lungs, and kidneys) [51], and cows (in the liver) [52]. These studies indicate that *SREBF2* plays critical roles in diverse tissues. In this study, we observed that *SREBF2* is also expressed in all 11 tissues tested of both lactating and dry-period buffalo. Notably, buffalo milk has a higher fat content than cow milk, suggesting metabolic differences between the mammary glands of the two types of animals [53,54]. A further analysis revealed that *SREBF2* expression in the buffalo mammary gland is higher in lactation than in the dry period, indicating that SREBF2 plays a role in lactating buffalo mammary glands. Previous research has also shown that *SREBF2* expression in dairy cow mammary glands is significantly upregulated during lactation (*p* < 0.05) compared to the dry period [45]. Interestingly, cholesterol synthesis in dairy cow mammary glands relies almost exclusively on de novo synthesis pathways [55] despite the low cholesterol levels in cow milk [56]. Studies have reported that the expression of genes related to cholesterol biosynthesis in dairy cow mammary tissue increased significantly (1.5- to 2-fold) during lactation [57]. These findings suggests that cholesterol synthesis becomes more active during lactation, with a corresponding upregulation of relevant genes. In addition, throughout the entire lactation period, this study observed fluctuations in *SREBF2* expression levels during the early lactation stage. The likely reason behind this phenomenon is closely related to the physiological state of the buffalo during this phase. Specifically, the early lactation period marks the beginning of the lactation cycle. To meet the demand for milk production, the lipid metabolism of buffalo is significantly enhanced. However, at this point, the energy requirements of buffalo have exceeded the energy provided by the feed intake. Given this energy imbalance, buffalo have to actively initiate the metabolism of stored lipids to compensate for the energy needed for milk production [58,59]. These complex physiological changes are highly likely to affect the expression levels of *SREBF2*, leading to the observed substantial fluctuations.

The PI3K/AKT/mTOR signaling pathway plays a crucial role in maintaining milk fat metabolism in dairy cows [60] and regulates the expression of genes involved in de novo milk fat synthesis in buffalo [61]. This pathway is initially activated through the phosphorylation of insulin receptor substrates (IRS-1 and IRS-2), which subsequently activates PI3K [62]. PI3K catalyzes the conversion of phosphatidylinositol-4,5-bisphosphate (PIP2) into PIP3 [63]. PIP3 interacts with AKT, leading to the activation of mTOR [64]. Activated mTOR stimulates the transcription factor SREBF1, which is cleaved into the nucleus to directly regulate the expression of *SREBF1* and *SREBF2* [65], forming the PI3K/AKT/mTOR/SREBFs signaling pathway [66]. In this study, *SREBF2* was knocked down in BuMECs, which significantly reduced the expression of key genes in the PI3K/AKT/mTOR pathway. In addition, a notable decrease in TAG and cholesterol levels was observed in BuMECs. These findings suggest that *SREBF2* knockdown impairs the regulatory capacity of the PI3K/AKT/mTOR pathway to regulate the expression of genes related to TAG and cholesterol synthesis, leading to decreased TAG and cholesterol synthesis and content.

SREBF1, PPARG, INSIG1, and INSIG2 are key regulators of lipid metabolism. In mice and humans, the *SREBF1* gene encodes both SREBF1a and SREBF1c isoform [67]. Functionally, the SREBF1c isoform is more selective for genes involved in fatty acid synthesis, while the SREBF1a isoform and SREBF2 primarily regulate cholesterol synthesis due to differences in their specificity for target gene promoters [68]. Previous studies have shown that small amounts of the *SREBF1a* isoform and *SREBF2*, when co-transfected into human embryonic kidney 293 cells, can activate the transcription of target genes containing *SRE* [1]. However, once the transfection reaches saturation, the co-activation of target gene transcription by SREBF1a and SREBF2 does not increase additively, indicating a threshold effect in their co-regulatory roles [1]. In regard to the interaction of SREBF1 and SREBF2, in the liver of *SREBF1*^−/−^ mice, fatty acid synthesis is reduced, and SREBF2 levels may increase to compensate for the loss of SREBF1 [3]. On the other hand, after we knocked down *SREBF2* in BuMECs, the level of cholesterol reduction was higher than the levels of TAG; this suggests that *SREBF1* provides limited compensation for the loss of *SREBF2*. Intracellular regulation of SREBFs occurs at both the transcriptional and post-transcriptional levels [69]. The post-transcriptional regulation described in the introduction involves the sterol-mediated inhibition of SREBF cleavage. The transcriptional regulation of SREBFs is more complex, as they can regulate their own expression. In addition, PPARG, a key regulator of fatty acid synthesis, contains *SRE* and *E-box* elements that can be specifically bound by SREBF2 [70,71]. In our study, inhibiting *SREBF2* reduced the expression of both *SREBF1* and *PPARG*. This suggests that *SREBF2* is involved in TAG and cholesterol biosynthesis in BuMECs by positively regulating the expression of genes encoding these factors through both direct and indirect mechanisms.

It is worth mentioning that the downregulation of *SREBF2* in BuMECs was associated with decreased *INSIG1* expression and increased *INSIG2* expression. Previous studies have shown that INSIG1 and INSIG2 can bind to SCAP in the endoplasmic reticulum, inhibiting the activation of SREBFs [72]. These two INSIG proteins exhibit complementary regulatory roles, with one compensating for a reduction in the other [73]. However, this hypothesis warrants further experimental validation. Additionally, *ACACA*, *SCD*, *HMGCR*, and *SQLE* all also contain *SREs* [74,75]. In the present study, downregulation of *SREBF2* in BuMECs led to reduced expression of *ACACA*, *SCD*, *HMGCR*, and *SQLE*, suggesting diminished binding of SREBF2 to these genes’ *SREs*, thereby lowering their expression. This highlights the critical role of SREBF2 in modulating TAG and cholesterol synthesis in BuMECs.

Amino acid sequence comparison revealed that the N-terminal acidic domain of SREBF2 in Bovidae species is particularly rich in proline, serine, glutamine, and glycine. Interestingly, asparagine is also concentrated in this domain in *Ovis*. Asparagine plays a crucial role in N-glycosylation, a modification essential for protein structure stability and function [76]. Furthermore, we identified the sites c.215G (p.72S) and c.2243A (p.748H), which distinguish *Bubalus* from *Bos* and *Ovis*. Sequence variations and their genetic analysis showed that synonym or non-synonym substitutions do not affect the function of buffalo *SREBF2*, which indicates that *SREBF2* is functionally conservative in two types of buffalo. Despite differences in the amino acid sequences of SREBF2 in *Bubalus*, *Bos*, and *Ovis*, the physicochemical properties of the different amino acids at the sites (e.g., p.206, p.214, p.748, and p.870) are similar. This suggests that the function of *SREBF2* is conserved across Bovidae species.

## 5. Conclusions

In this study, we successfully cloned the CDS of *SREBF2* from the buffalo mammary gland and demonstrated its high functional similarity across Bovidae species. Our findings highlight the critical regulatory role of SREBF2 in the mammary gland during lactation, particularly in regulating TAG and cholesterol biosynthesis both directly and indirectly via the PI3K/AKT/mTOR/SREBF2 signaling pathway. Additionally, SREBF2 was found to promote TAG biosynthesis by directly upregulating key transcription factors, including SREBF1 and PPARG. A total of 22 SNPs were identified in both buffalo species, and the assessment showed that these SNPs did not affect the functionality of *SREBF2*. Whether these SNPs have any influence on the lactation traits of buffalo needs to be further studied. Overall, this study provides valuable insights into the molecular characteristics and functional role of the buffalo *SREBF2* gene, particularly its involvement in TAG and cholesterol biosynthesis in BuMECs.

## Figures and Tables

**Table 1:** The polymorphic sites and allele frequencies, *p* value, and expected heterozygosity of the *SREBF2* gene in river and swamp buffalo.

Population	Location	SNPs	Genotype Number			Gene Frequency		*p* Value ^1^	Expected Heterozygosity
			WW	Wm	mm	W	m		
River buffalo	Exon1	c.477G>A	43	22	0	0.830769	0.169231	0.576646	0.260400
	Exon2	c.636C>G	49	16	0	0.876923	0.123077	0.632585	0.277800
	Exon3	c.795G>A	49	16	0	0.876923	0.123077	0.632585	0.277800
	Exon5	c.1095T>C	49	11	5	0.838462	0.161538	0.077074	0.260400
	Exon9	c.2017C>T	54	11	0	0.915385	0.084615	0.796253	0.197500
	Exon10	c.2079C>T	49	5	11	0.792308	0.207692	0.015075	0.401200
	Exon10	c.2082T>A	54	0	11	0.830769	0.169231	0.000701	0.345700
	Exon12	c.2475A>G	54	0	11	0.830769	0.169231	0.000042	0.260400
	Exon15	c.2799C>T	65	0	0	1.000000	0.000000	1.000000	0.104900
	Exon17	c.3186C>T	49	16	0	0.876923	0.123077	0.632585	0.277800
	Exon18	c.3354G>A	59	6	0	0.953846	0.046154	1.000000	0.104900
Swamp buffalo	Exon1	c.246C>T	51	14	0	0.892308	0.107692	0.218800	0.197500
	Exon3	c.843C>T	7	0	58	0.107692	0.892308	0.218800	0.197500
	Exon6	c.1240G>A	43	22	0	0.830769	0.169231	0.304700	0.277800
	Exon10	c.2082T>A	51	7	7	0.838462	0.161538	0.117200	0.277800
	Exon13	c.2505C>T	51	14	0	0.892308	0.107692	0.304700	0.277800
	Exon15	c.2799C>T	51	14	0	0.892308	0.107692	0.218800	0.197500
	Exon15	c.2802G>T	58	7	0	0.946154	0.053846	0.218800	0.197500
	Exon16	c.2944G>A	58	7	0	0.946154	0.053846	0.117200	0.104900
	Exon17	c.3186C>T	43	22	0	0.830769	0.169231	0.304700	0.277800
	Exon18	c.3354G>A	36	29	0	0.776923	0.223077	0.375000	0.345700

^1^ *p* value of the Hardy–Weinberg equilibrium test.

## Data Availability

The raw data supporting the conclusions of this article will be made available by the authors on request.

## Supplementary Materials

The following supporting information can be downloaded at https://www.mdpi.com/article/10.3390/genes16020237/s1, Figure S1. The original picture of BuMECs; Figure S2: PCR gel electrophoresis of buffalo *SREBF2* CDS. M, Marker-DL2000; 1 and 2, PCR amplification results; Figure S3: CDS nucleotide and its coding protein sequence of buffalo *SREBF2* gene; the highlighted regions indicate the bHLH domains (amino acids [AAs] 297–347); Figure S4: Consistency of SREBF2 protein sequences in 13 mammalian species, including buffalo. The red above the gray diagonal represents sequence identity, while the blue below the diagonal indicates sequence divergence. Figure S5: Phylogenetic relationships, motif composition, and conserved domains. (A) Phylogenetic tree constructed based on SREBF2 sequences from 13 mammalian species. (B) Motif composition of SREBF2 across different species. (C) Conserved domains of SREBF2 in 13 mammals. Colored boxes indicate distinct motifs and conserved domains. Figure S6: Functional modification sites of SREBF2 proteins in buffalo and other Bovidae species. Figure S7: Protein–protein interaction network of buffalo SREBF2. The colors of the lines represent different patterns of predicted interactions. Light green is used for text mining, while red is used for gene fusion, purple is used for experimental determination, and cyan is used for achievements from the curated database. Figure S8: Differences in *SREBF2* nucleotide sequences between buffalo and other Bovidae species, with numbers representing base positions. A dot (.) indicates identity with *SREBF2*, while nucleotide substitutions are represented by different letters. Question marks (?) are used for missing information markers. Horizontal lines (-) represent deletion in the sequences. Table S1: The information of the primers utilized in this study. Table S2: Secondary structure of the Bovidae species SREBF2. Table S3: Physicochemical properties of SREBF2 proteins from buffalo and other mammals. Table S4: Relative synonymous codon usage of the Bovidae species *SREBF2*. Table S5: The haplotypes of the buffalo *SREBF2* gene and their frequencies.
