# Supplementary material for: Molecular Characteristics and Role of Buffalo SREBF2 in Triglyceride and Cholesterol Biosynthesis in Mammary Epithelial Cells
Source: Genes (Basel). 2025 Feb 19;16(2):237. doi: 10.3390/genes16020237 (PMC11855135; doi:10.3390/genes16020237)
Supplement: Supplementary file 1 [file genes-16-00237-s001.zip › Figure S1-S5.pdf]

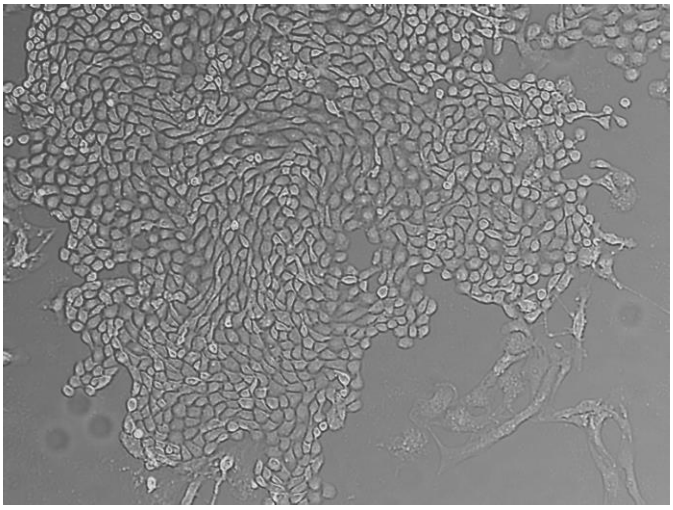

Figure S1. The original picture of BuMECs.

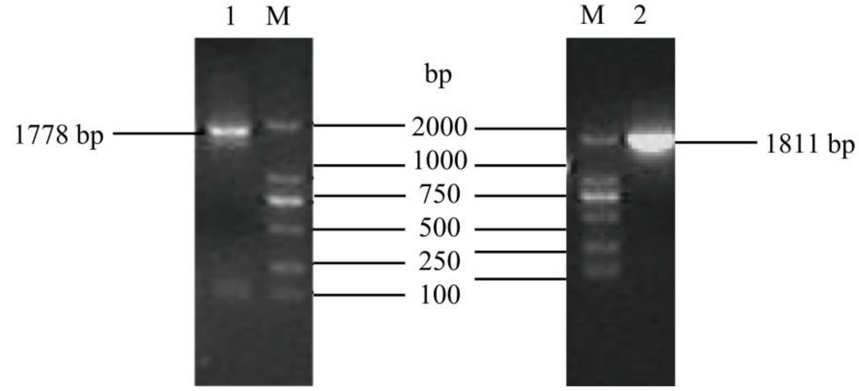

Figure S2. PCR gel electrophoresis of buffalo *SREBF2* CDS. M, Marker-DL2000; 1 and 2, PCR amplification results.

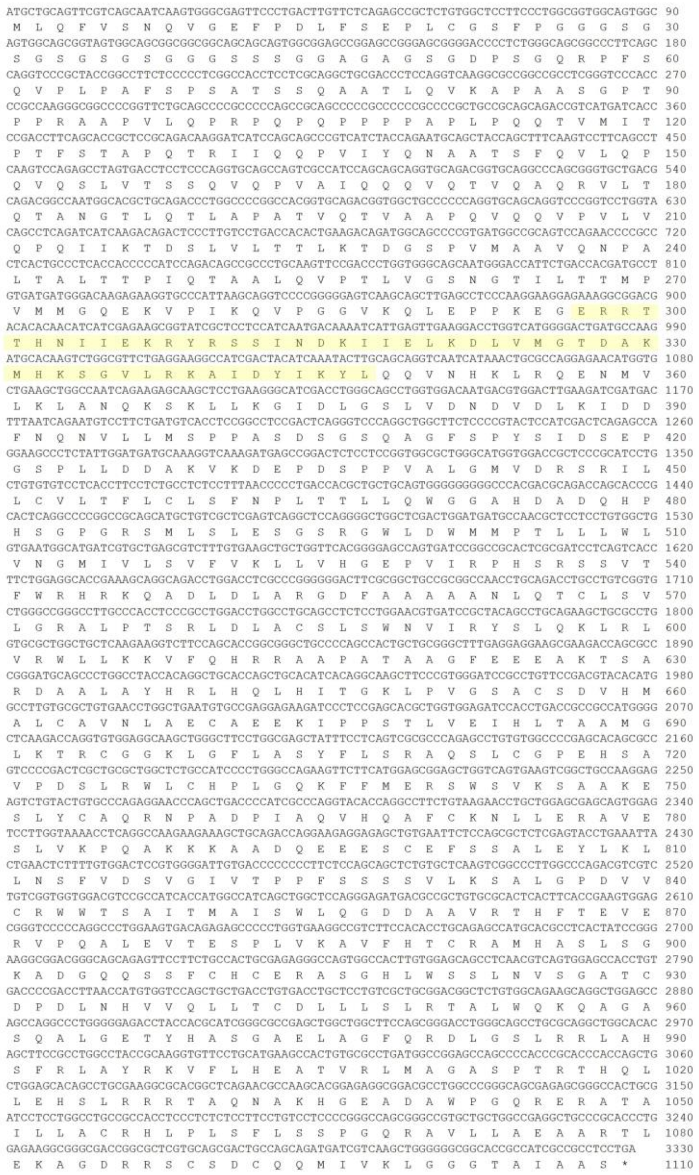

Figure S3. CDS nucleotide and its coding protein sequence of buffalo *SREBF2* gene; The highlighted parts are the bHLH domains (amino acids [AAs] 297-347).

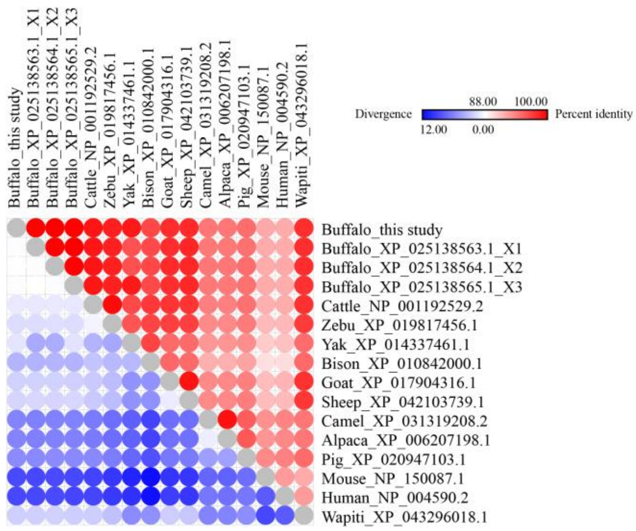

Figure S4. Consistency of SREBF2 protein sequences in 13 mammalian species including buffalo. The red above the gray diagonal represents sequence identity, while the blue below the diagonal indicates sequence divergence.

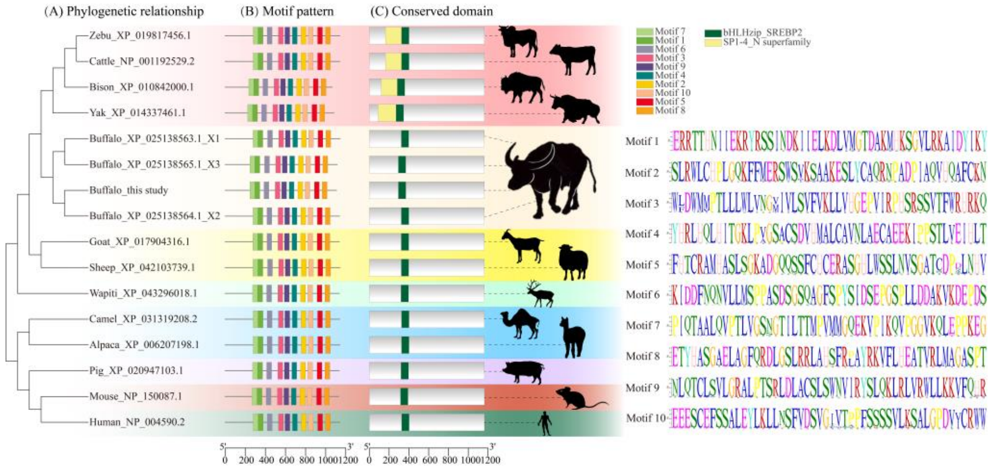

Figure S5. Phylogenetic relationships, motif composition, and conserved domains. (A) Phylogenetic tree constructed based on SREBF2 sequences from 13 mammalian species. (B) Motif composition of SREBF2 across different species. (C) Conserved domains of SREBF2 in 13 mammals. Colored boxes indicate distinct motifs and conserved domains.

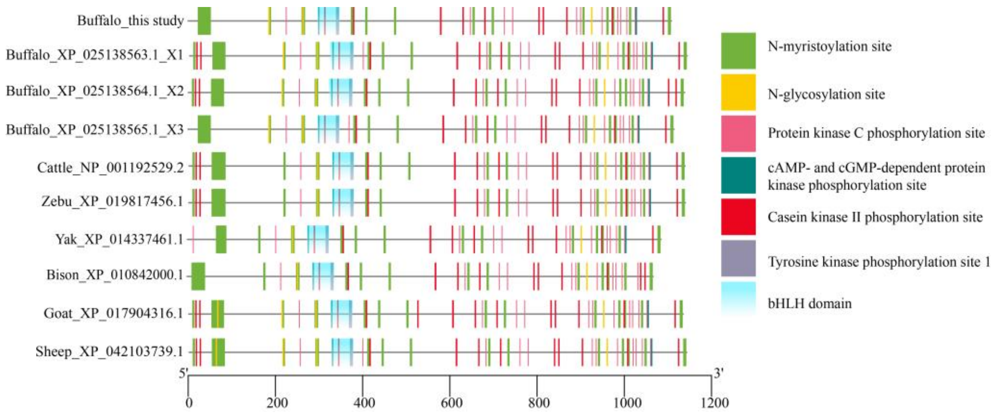

Figure S6. Functional modification sites of SREBF2 proteins in buffalo and other Bovidae species.

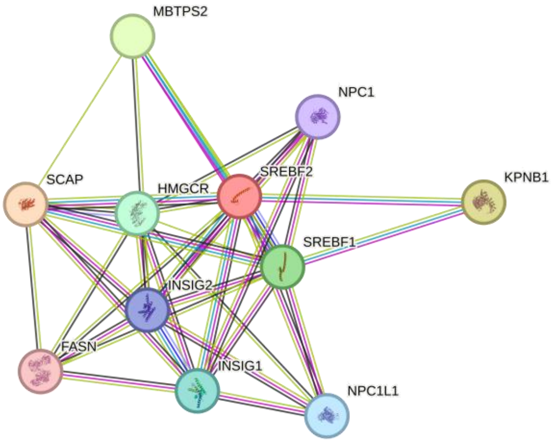

Figure S7. Protein–protein interaction network of buffalo SREBF2. The colors of the lines represent different patterns of predicted interactions. Light green is used for text mining, while red is used for gene fusion, purple is used for experimental determination, and cyan is used for achievements from the curated database.

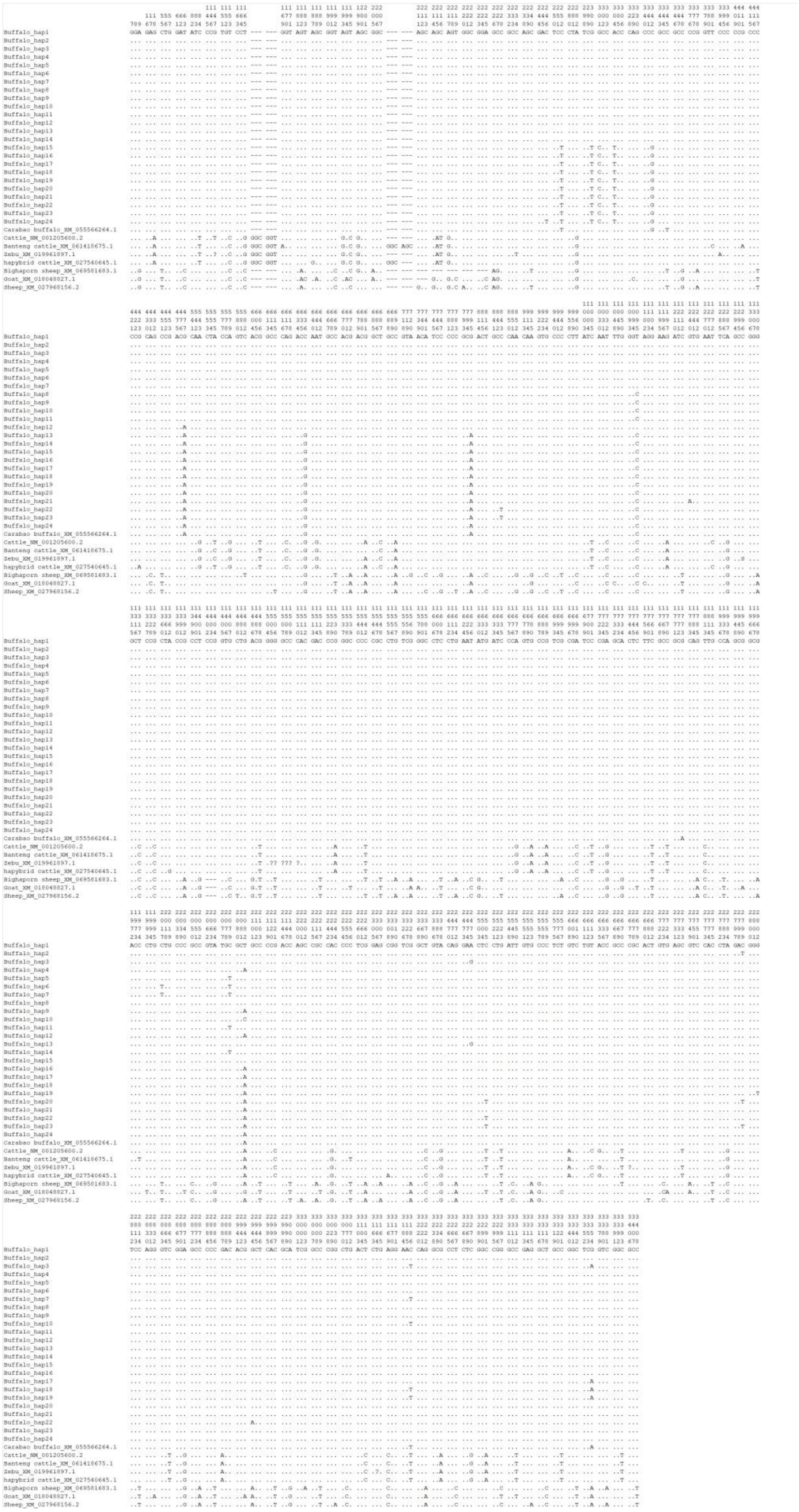

Figure S8. Differences in *SREBF2* nucleotide sequences between buffalo and other Bovidae species, with numbers representing base positions. A dot (.) indicates identity with *SREBF2*, while nucleotide substitutions are represented by different letters. Question marks (?) are used for missing information markers. Horizontal lines (-) represent deletion in the sequences.
